# Effect of aflatoxin B_1_ on blood serum oestradiol-17β and progesterone concentrations during the luteal phase and the synchronized oestrus of goats

**DOI:** 10.21451/1984-3143-2017-AR939

**Published:** 2018-08-16

**Authors:** Georgios D. Kourousekos, Ekaterini K. Theodosiadou, Aristotelis G. Lymberopoulos, Sofia Belibasaki, Constantin Boscos

**Affiliations:** 1 Faculty of Veterinary Medicine, , , .; 2 Faculty of Veterinary Medicine, University of Thessaly, 43100, Karditsa, .; 3 Department of Animal Production, , , .; 4 Veterinary Research Institute, , , .

**Keywords:** mycotoxins, AFB_1_, reproduction, ruminants, hormones

## Abstract

The effect of prolonged aflatoxin B_1_ (AFB_1_) administration on blood serum oestradiol-17β and progesterone concentrations in goats during the luteal phase and the synchronized oestrus was investigated. Thirty-six Greek indigenous primiparous goats were used, during the oestrus period; 12 goats received, per os, 50 μg (treated group T50) and 12 goats received 100 μg (treated group T100) AFB_1_/day/head, respectively, for approximately 1.5 month, while 12 goats served as controls (C). On day 36 of the experiment, each goat was injected, i.m, 0.5 ml prostaglandin F_2α_ (PGF_2α_). Blood samples were collected from each goat twice a week, before PGF_2α_ injection, as well as every 4 hours from the onset to the end of the synchronized oestrus. Oestradiol-17β and progesterone concentrations in blood serum were determined using radioimmunoassay. During the whole luteal(s) phase(s), linear regression analysis revealed a significant negative dependence (P < 0.05) of oestradiol-17β and a significant positive dependence (P < 0.05) of progesterone over group (C = 0, T50 = 50, T100 = 100), in a dose dependent manner. During the synchronized oestrus, multiple linear regression analysis revealed a significant negative dependence (P < 0.05) of oestradiol-17β, as well as a significant positive dependence (P < 0.05) of progesterone over group (C = 0, T50 = 50, T100 = 100) and over time (hours, from the onset to the end of the synchronized oestrus). No significant differences were noticed among the three groups, regarding the body weight of the goats from the onset to the end of AFB_1_ administration, the occurrence or the duration of the synchronized oestrus presented by the goats (P > 0.05). In conclusion, prolonged AFB_1_ administration at doses of 100 or even of 50 μg/day/head changes the hormonal pattern in blood during the luteal phase and the synchronized oestrus of goats, being in oestrus period.

## Introduction

Aflatoxins (B_1_, B_2_, G_1_ and G_2_) are toxic metabolites produced by the fungi *Aspergillus flavus* and *Aspergillus parasiticus* and considered carcinogenic for animals and humans ( Klich, 2007 ). AFB_1_ is very regularly detected in feedstuffs and sometimes in feed from households ( Klarić *et al*., 2009 ) causing various detrimental effects after it is consumed by animals or humans. Furthermore, aflatoxin Μ_1_ (AFM_1_), the hydroxylized metabolite of AFB_ 1_, is excreted into the milk of most animal species and of human, as well ( Galvano *et al*., 1996 ; Abdulrazzaq *et al*., 2003 ) and considered carcinogenic.

The mechanisms, through which AFB_1_ affects the reproductive system, remain unexplained, since the effects of the toxin have not been studied extensively ( Shuaib *et al*., 2010 ). Aflatoxins are easily and rapidly absorbed from both the gastrointestinal tract and through the peritoneum as Bastaki *et al*. (2010) observed after administrating 20 mg AFB_1_/kg body weight, orally or intraperitoneally on gestation day 13 in pregnant mice. After AFB_1_ administration (7.5 mg/kg body weight/day for 14 days) in female rats, inhibition of oocytes growing, reduction of the ovary size and weight, reduced oestradiol-17β concentration and increased progesterone concentration in blood ( Ibeh and Saxena, 1997a ) were observed. During rabbits’ gestation, a considerable decrease in fetal weight was recorded, after AFB_1_ was administered at 0.1 mg/kg body weight *per os* ( Wangikar *et al*., 2005 ). Moreover, the high risk of AFB_1_ toxicity on the foetus, involve aflatoxicol production, an AFB_1_ metabolite with carcinogenic potency, from the placenta of the women studied ( Partanen *et al*., 2010 ); with that risk being more serious in high-risk countries, such as Egypt ( Piekkola *et al*., 2012 ).

Recently, Storvik *et al*. (2011) indicated that the chronic exposure to AFB_1_ might cause endocrine disruption in the human foetoplacental unit due to its effect on the expression of aromatase enzymes (P450s or CYPs enzymes), categorizing AFB_1_ as a potential endocrine disruptor. Endocrine disruptors could affect steroid ovarian hormones concentrations, either directly or indirectly. The alterations in oestradiol-17β and/or progesterone concentrations during the luteal phase and/or the synchronized oestrus may have detrimental effects on subsequent reproductive life of the animals, such as shortened cycles, lower fertility, negative effects on follicle maturation, ovulation or the presence and/or the signs of the oestrus cycle.

Specifically in goats, the reproductive effects of AFB_1_ administration are limited. Furthermore, the studies that have already been performed, mainly in laboratory animals, use quite higher concentrations and/or shorter periods of AFB_1_ administration. In a previous study ( Kourousekos *et al*., 2015 ) the prolonged administration of 100 or even of 50 μg AFB_1_/day/head increased blood serum oestradiol-17β and progesterone concentrations in anoestrous goats, in a dose dependent manner. Thus, the present study was conducted in order to investigate the possible effects by AFB_1_ administration on blood serum oestradiol-17β and progesterone concentrations, during the luteal phase and the synchronized oestrus and consequently on ovarian activity and oestrus cycle of goats being in oestrus period.

## Materials and Methods

### Animals and experimental protocol

For the aim of the present study 36 Greek indigenous primiparous goats, 2-3 years old, weighting 32.6 ± 2.4 kg, housed in open-fronted covered yard and being at the oestrus period were used. The study was conducted at the Veterinary Research Institute, in northern Greece, from middle of September to middle of November (longitude 22^o^51´37´´E and latitude 40^o^41´19´´N; average temperature 17.7 ^o^C and average humidity 62.7%; day-length 11.2 hours with 12.8 hours darkness). All goats performed parturition about seven months ago without their oestrus cycles previously being synchronized. Throughout the experimental period, the goats were not in contact with other animals, were healthy and no pharmaceutical treatment were given to them. The goats were fed 1 kg/day/head of pelleted concentrate plus grass hay *ad libitum* ; water was available for the animals 24 hours a day. The pelleted concentrate feed consisted of corn, barley, gluten, soybean meal, molasses, yeast ranching, sodium chloride, calcium carbonate, dicalcium phosphate, vegetable fat, vitamins and minerals. The chemical analysis of the pelleted concentrate feed was: dry matter 52.4%, total proteins 17.2%, fat 3.3%, cellulose 3.9%, ashes 7.7%, humidity 13.0%, calcium 1.2%, phosphorus 0.7%, sodium 0.6% and chlorine 0.04%.

To ensure that the goats did not receive any AFB_1_ concentrations through their diet, feedstuff samples were analyzed, once a week, for AFB_1_ presence using high performance liquid chromatography (HPLC), as described by Akiyama *et al*. (2001) with the assistance of specific columns (MycoSep^®^ 226 columns for AFB _1_). The limit of detection for AFB_1_ was 0.5 μg/kg. None of the samples was found positive in AFB_1_ presence.

Goats were randomly divided into 3 groups of 12 animals each [control group (C), treated group (T50) and treated group (T100)]. The experiment lasted approximately 1.5 month, during which the goats of T50 or T100 groups received 50 or 100 μg AFB_1_/day/head, respectively [doses by which AFM_1_ is excreted into the milk, exceeding the maximum permissible level (50 ng/L) set by the European Union ( Kourousekos *et al*., 2012 )]. Ten mg of pure AFB_1_ (AFB_1_ from *Aspergillus flavus* , A 6636-10 MG, SIGMA, Sigma Chemical Co, St. Louis, MO, USA) were dissolved in 100 or 200 mL methanol and 1 mL of this dilution was received *per os* by each goat of T50 or T100 treated groups, respectively. The goats of the control group received only the solvent of AFB_ 1_ (1 mL methanol/day) in order to be equally handled; methanol is the best solvent for AFB_1_ for *in vivo* treatment according to Battacone *et al*. (2003) , while the administration of methanol at these concentrations has no risk for the animal’s health ( http://www.epa.gov/chemfact/s_methan.txt ). The administration of the diluted AFB_1_ in the goats of T50 or T100 treated groups, as well as the administration of 1 mL methanol in the goats of group C was achieved, at the same hour (07:00 a.m.) every morning, using a dosimetric pistollete-like pump, in order to be controlled and easily accepted.

On day 36 of the experiment, 0.5 ml PGF_2α_ (Estrumat®, Schering-Plugh, USA) were injected, i.m., in each goat for the synchronization of their oestrus cycles. In the goats where no oestrus was detected after 96 hours from the first injection, 0.5 ml PGF_ 2α_ was injected for a second time after an 11-day interval.

From the beginning of the experiment and before PGF_2α_ injection, blood samples were collected twice a week from each goat (always at the same days and at the same time about 08:00 a.m.). After PGF_2α_ injection blood samples were collected from each goat at 4 hours intervals from the onset to the end of the synchronized oestrus. Oestrus detection was realized, every 6 hours, by three teaser bucks. Blood samples were collected by jugular venipuncture into evacuated blood collecting tubes (Venoject, Terumo, Belgium). After clotting, blood samples were centrifuged (2500 x g; 20 min; 4^o^C); serum was aspirated and stored at –20^o^C until assayed. Moreover, the body weight (kg) of all goats was measured once a week.

### Oestradiol-17β and progesterone assays

Oestradiol-17β and progesterone concentrations in blood serum were determined, in duplicate, using radioimmunoassay (RIA), after extraction, as described by Martin *et al*. (1987) , following minor modification. The radiolabelled solutions of oestradiol-17β and progesterone were provided by Amersham Biotech, (Buckinghamshire, UK), while oestradiol-17β and progesterone antiserums, were developed by the Institute of Molecular Biology, Iraklion, Crete, Greece ( Theodosiadou *et al*., 2004 ). The sensitivity (lower limit of detection) for oestradiol-17β was 3.90 pg/mL, while for progesterone was 19 pg/mL (0.019 ng/mL). The intra-assay variability was 3.4-6.0% (n = 8) and 2.8-4.8% (n = 8), while the inter assay variability was 9.5% (n = 72) and 8.5% (n = 72) for oestradiol-17β and progesterone, respectively. The recovery rate was estimated to be 88.3% ± 3.4% (Mean ± SD; n = 72) and 90.5% ± 2.4% (Mean ± SD; n = 72) for oestradiol-17β and progesterone, respectively.

### Statistical analysis

One-way analysis of variance (one-way ANOVA) was used to compare the body weight, the occurrence and the duration of the synchronized oestrus, as well as blood serum oestradiol-17β or progesterone concentrations among the three groups studied. Levene’s test was used for the control of homogeneity of variances and statistical differences were estimated using Tukey’s HSD test. Linear regression analysis was used in order to trace the variability of oestradiol-17β or progesterone concentration over group (C = 0, T50 = 50, T100 = 100) and over time (in days for the luteal phase, or in hours for the synchronized oestrus) (multiple), or over group, or over time. Statistical analysis was performed using SPSS^®^ software (Version 15.0, 2006, SPSS Inc., Athens, Greece) for MS Windows; in all cases, a probability of P < 0.05 was the minimum level of significance.

## Results

### Body weight (kg)

No significant differences (P > 0.05) were observed among the three groups studied (C: 33.6 ± 1.9; T50: 32.8 ± 2.5; T100: 31.5 ± 2.9; Mean ± SD) during the whole experimental period.

### Luteal phase

**Figure 1:**
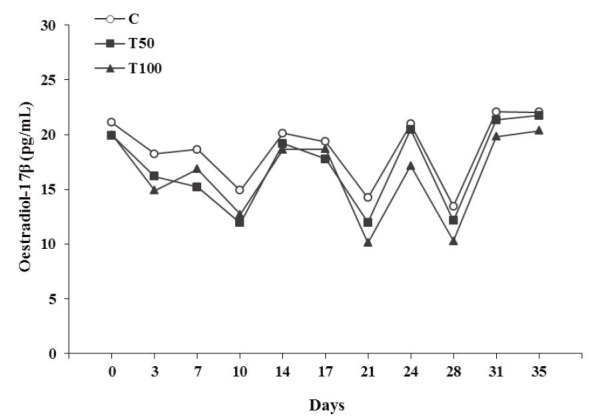
Oestradiol-17β concentration in blood serum (pg/mL) during the luteal(s) phase(s) of the goats [control group (C); treated group T50 (50 μg AFB1/goat/day); treated group T100 (100 μg AFB1/goat/day); (Mean ± SD)].

**Figure 2:**
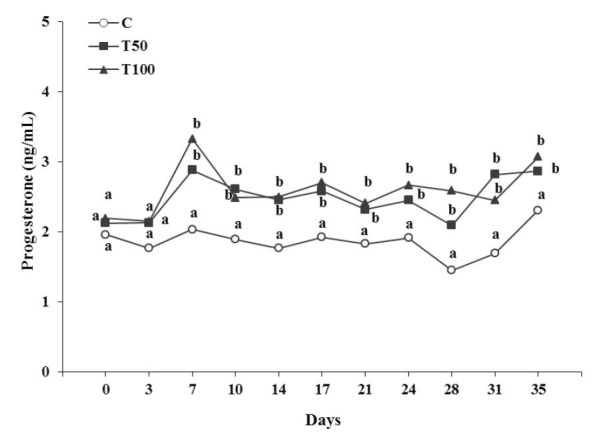
Progesterone concentration in blood serum (ng/mL) during the luteal(s) phase(s) of the goats [control group (C), treated group T50 (50 μg AFB1/goat/day); treated group T100 (100 μg AFB1/goat/day); (Mean ± SD)]. ^a,b,c^ Significant differences among the three groups at each time point studied (P < 0.05).

During the whole luteal(s) phase(s), oestradiol-17β concentration of the treated groups T50 or T100 presented significantly lower (P < 0.05), while progesterone concentration presented significantly higher (P < 0.05) than those of the goats of group C ( Figs. 1 - 2 ). Analytically, at the first 3 days after AFB_1_ administration, progesterone concentration showed no significant differences (P > 0.05) among the three groups studied. From 7 to 35 days after AFB_1_ administration progesterone concentration of the treated groups T50 or T100 presented significantly higher (P < 0.05) compared to that of group C, while no significant difference (P > 0.05) was observed between T50 and T100 groups ( Fig. 2 ).

More specifically, linear regression analysis revealed a significant decrease of oestradiol-17β concentration (F = 9.98, df = 306, P = 0.002; Constant = 19.67 ± 0.81, t = 24.38, P = 6.94E-021; Group = -1.16 ± 0.37, t = -3.16, P = 0.002), as well as, a significant increase of progesterone concentration (F = 66.62, df = 306, P = 8.29E-015; Constant = 1.59 ± 0.09, t = 16.39, P = 6.94E-021; Group = 0.36 ± 0.04, t = 8.16, P = 8.29E-015) over group (C = 0, T50 = 50, T100 = 100), in a dose dependent manner.

The luteal phase was defined by progesterone concentrations >1 ng/mL and oestradiol-17β concentrations <27.2 pg/mL ( Chemineau et al., 1982 ; Bauernfeind and Holtz, 1991 ). All other values during the period of the 35 days treatment were excluded, since those values represented the natural oestrus of the goats.

### Synchronized oestrus

After the first PGF_2α_ injection, oestrus was detected in 26 goats (C: n = 9; T50: n = 8; T100: n = 9). After the second PGF_2α_ injection (in the goats that did not respond to the first injection) oestrus was detected in another 8 goats (C: n = 2; T50: n = 3; T100: n = 3). Two goats did not respond at any PGF_2α_ injection and excluded from the study. No significant differences were observed among the three groups studied (P > 0.05), regarding the number of goats that responded to PGF_2α_ injection.

Regarding the duration of the synchronized oestrus that followed either the first or the second PGF_2α_ injection, no significant differences were observed among the three groups studied (P > 0.05). The mean oestrus duration in hours (Mean ± SD) was 38.9 ± 5.0 (n = 11), 36.7 ± 6.6 (n = 11) and 38.3 ± 6.7 (n = 12) for group C, T50 and T100, respectively.

**Figure 3:**
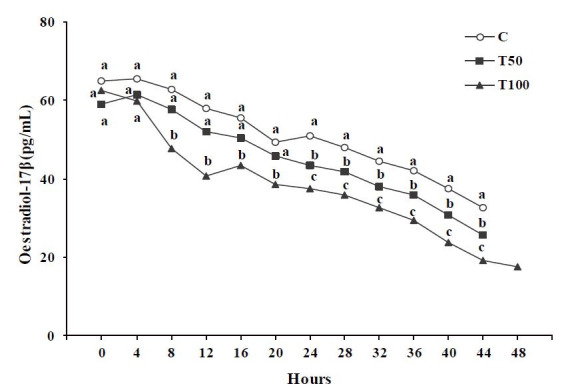
Oestradiol-17β concentration in blood serum (pg/mL) during the synchronized oestrus of the goats [control group (C); treated group T50 (50 μg AFB1/goat/day); treated group T100 (100 μg AFB1/goat/day); (Mean ± SD)]. ^a,b,c^ Significant differences among the three groups at each time point studied (P < 0.05).

During the synchronized oestrus, oestradiol-17β concentration of the goats of the treated groups T50 or T100 presented significantly lower (P < 0.05), than that of group C ( Fig. 3 ). Analytically, at the first 4 hours from the onset of the synchronized oestrus no significant differences were observed among the three groups studied (P > 0.05). From 8 to 20 hours oestradiol-17β concentration of the goats of T100 group presented significantly lower (P < 0.05) than that of the goats of T50 or C group, while no significant difference was observed between T50 and C groups (P > 0.05). From 24 hours until the end of the synchronized oestrus oestradiol-17β concentration was significantly lower (P < 0.05) at the goats of the treated groups T50 or T100 compared to that of group C; the lowest concentration was observed in T100 group ( Fig. 3 ).

Specifically, multiple linear regression analysis revealed a significant decrease of oestradiol-17β concentration over group (C = 0, T50 = 50, T100 = 100) and over time (hours from the onset to the end of the synchronized oestrus) (F = 285.49, df = 355, P = 0.0; Constant = 74.12 ± 1.43, t = 51.99, P = 3.86E-021; Group = –5.79 ± 0.56, t = –10.28, P = 3.86E-021; Time = –0.79 ± 0.04, t = –21.65, P = 3.86E-021).

**Figure 4:**
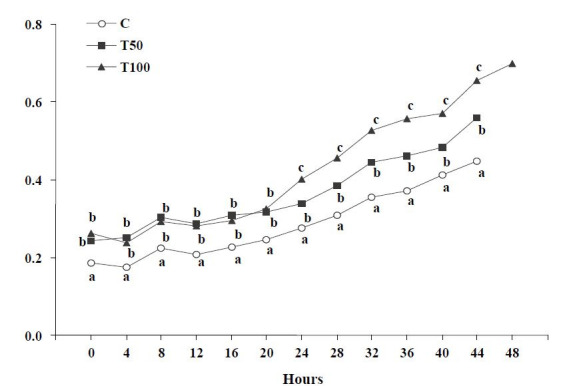
Progesterone concentration in blood serum (ng/mL) during the synchronized oestrus of the goats [control group (C); treated group T50 (50 μg AFB1/goat/day); treated group T100 (100 μg AFB1/goat/day); (Mean ± SD)]. ^a,b,c^ Significant differences among the three groups at each time point studied (P < 0.05).

Progesterone concentration of the goats of the treated groups T50 or T100 presented significantly higher (P < 0.05) than that of group C ( Fig. 4 ). Analytically, from the onset to 20 hours of the synchronized oestrus progesterone concentration of the goats of the treated groups T50 or T100 presented significantly higher (P < 0.05) than that of the goats of group C, while no significant difference was observed between T50 and T100 groups (P > 0.05). From 24 hours until the end of the synchronized oestrus progesterone concentration remained significantly higher (P < 0.05) at the goats of the treated groups T50 or T100 compared to that of group C; the highest concentration was observed in T100 group ( Fig. 4 ).

Specifically, multiple linear regression analysis revealed a significant increase of progesterone concentration over group (C = 0, T50 = 50, T100 = 100) and over time (hours from the onset to the end of the synchronized oestrus) (F = 315.09, df = 355, P = 0.0; Constant = 0.08 ± 0.01, t = 6.26, P = 1.15E-09; Group = 0.06 ± 0.01, t = 11.13, P = 3.86E-021; Time = 0.01 ± 0,0003, t = 22.58, P = 3,86E-021). The decrease of oestradiol-17β concentration and the increase of progesterone concentration, over time (hours from the onset to the end of the synchronized oestrus), in each group, are presented in Table 1 .

**Table 1:** Linear regression analysis results showing the negative or the positive dependence of blood serum oestradiol-17β or progesterone concentration over time (hours from the onset to the end of the synchronized oestrus) of the goats, in each group studied [control group (C); treated group T50 (50 μg AFB_1_/goat/day); treated group T100 (100 μg AFB_1_/goat/day)].

Oestradiol-17β (pg/mL)								Progesterone (ng/mL)						
		df	*F*	Sign. *F*	Time	Constant				df	*F*	Sign. *F*	Time	Constant
C		117	122.52	4.78E-019	-0.72	67.01	C		117	126.58	4.78E-019	0.006	0.15
	Standard error				0.07	1.55		Standard error				0.001	0.01
	*t*				-11.07	43.32		*t*				11.25	12.39
	Significance of *t*				4.78E-019	4.78E-019		Significance of *t*				4.78E-019	4.78E-019
T50		110	149.56	6.39E-019	-0.77	62.14	T50		110	149.47	6.38E-019	0.006	0.22
	Standard error				0.06	1.42		Standard error				0.001	0.01
	*t*				-12.23	43.82		*t*				12.26	19.02
	Significance of *t*				6.39E-019	6.39E-019		Significance of *t*				6.38E-019	6.38E-019
T100		125	200.59	3.52E-019	-0.87	58.30	T100		125	284.26	3.52E-019	0.009	0.20
	Standard error				0.06	1.44		Standard error				0.001	0.01
	*t*				-14.16	40.37		*t*				16.86	15.06
	Significance of *t*				3.52E-019	3.52E-019		Significance of *t*				3.52E-019	3.52E-019

## Discussion

The results of the present study are confirmed by those of other researchers, but in different species. Ibeh and Saxena (1997a ; b) administered in female rats 7.5 mg AFB_1_/kg body weight/day for 14 days or 15 mg AFB_1_/kg body weight/day for 21 days, respectively, and observed that blood oestradiol-17β and progesterone appeared significantly lower and higher, respectively, in rats receiving aflatoxin. The authors proposed either a direct effect of AFB_1_ on ovarian secreting cells or on the hypothalamus-hypophysis-ovaries axis. In the present study, AFB_1_ was administered in quite lower doses, but for a longer period, and that could have influenced the ovarian activity or the above-mentioned axis. In the study of Hasanzadeh *et al*. (2011) the hormonal pattern was investigated in male rats after the administration of 0.8, 1.6 and 3.2 ppm AFB_1_ orally for 48 days. The concentrations of blood serum LH, testosterone and oestradiol-17β were significantly lower in the group of rats receiving the highest dose of AFB_1_. The authors proposed either a direct effect of AFB_1_ on testes secreting cells or on the hypothalamus-hypophysis-testes axis. Furthermore during the anoestrus period of the goats the prolonged *per os* administration of 100 or even of 50 μg AFB_1_/day/head increased blood serum oestradiol-17β and progesterone concentrations, in a dose dependent manner ( Kourousekos *et al*., 2015 ).

The similar chemical structure between aflatoxins and oestradiol-17β triggered some researchers to study the possible oestrogenic action of aflatoxins. Kyrein (1974) observed that aflatoxins B_1_, G_1_ and G_2_ did not present any binding affinity to oestrogen receptors derived from healthy uterus of calves. On the contrary, AFM_1_ presented an extent of receptors binding, although in quite higher concentrations. Furthermore, aflatoxicol, another AFB_1_ metabolite, showed a small binding ability to oestrogen receptors ( Blankenship *et al*., 1982 ). In our study, the possible binding of oestrogen receptors by AFB_1_ metabolites could have disturbed gonadotrophins secretion and consequently reduced oestrogen production.

Recently, Storvik *et al*. (2011) categorized AFB_1_ as a potential endocrine disruptor. It is known that AFB_ 1_ is metabolized by cytochrome P450 (CYPs) enzymes ( Buhler and Wang-Buhler, 1998 ). Furthermore, Storvik *et al*. (2011) and Huuskonen *et al*. (2013) supported that AFB_1_ increases the expression of CYP19A1 in human placenta cells. More specifically, Huuskonen *et al*. (2013) indicated that AFB_1_ affected the placental steroid hormone synthesizing, metabolizing and conjugating enzymes and that these alterations may lead to anomalies in the foetoplacental hormonal homeostasis, while Storvik *et al*. (2011) suggested that AFB_1_, after being metabolized in aflatoxicol, had effects on genes important in endocrine regulation in placental cells. Furthermore, since CYPs have been found to take part in steroid hormones synthesis, the increase of the expression of such enzyme by AFB _1_ in the placenta could result in increased progesterone production. In our study the increased blood serum progesterone concentration, in a dose dependent manner, might have been a result of such alterations of CYPs enzymes on the ovaries due to prolonged AFB_1_ administration.

At this point, the study of the effects of high progesterone or low oestradiol-17β concentrations during the oestrus period would be particularly useful. Menchaca and Rubianes (2001) supported that premature progesterone exposure early in the ovulatory cycle of the goat affected its length inducing short or shortened cycles. In the study of Theodosiadou *et al*. (2004) the synchronization of oestrus by administration of the standard dose of progesterone resulted in a decrease/increase of oestradiol-17β/progesterone, respectively, in blood plasma and oviductal wall, compared to natural oestrus. Regarding low oestradiol-17β concentrations, Gilad *et al*. (1993) reported that in cows with low plasma oestradiol-17β, the mean and basal concentrations and amplitudes of gonadotrophins were significantly lower by heat stress compared to cows with high plasma oestradiol-17β concentrations. Furthermore, Theodosiadou *et al*. (2014) revealed that at artificial insemination time (performed either at fixed-time or after oestrus detection in synchronized ewes), pregnant ewes had lower progesterone and higher oestradiol-17β concentrations in blood serum, and lower electrical resistance values in the cervical mucus (ERCM) than the non-pregnant ones. The increased progesterone concentrations in the periestrual period might have negatively affected spermatozoa, causing pregnancy failure, since a significant positive relation between progesterone concentrations and ERCM values was found. Moreover, Theodosiadou and Tsiligianni (2015) observed that blood serum progesterone concentrations and ERCM values at oestrus were lower in synchronized ewes that conceived after they were mated to fertile rams compared to those that did not conceive, either at oestrous or at anoestrous period. Finally, Carter *et al*. (2010) mentioned that elevated concentration of progesterone do not affect the ability of the early cow embryo to reach the blastocyst stage *in vivo*, but do result in subtle changes to the transcriptome of the embryo, due to advanced elongation post-hatching. Taking into account the above mentioned studies, the decreased/increased blood serum oestradiol-17β/progesterone concentrations, observed in our study, may cause detrimental effects on fertilization, conception and/or embryo development.

In conclusion prolonged AFB_1_ administration in goats, being at oestrus period, at the doses of 100 or even of 50 μg/day/head could lead to a decrease/ increase of blood serum oestradiol-17β/progesterone concentrations during the luteal phase and during the synchronized oestrus, in a dose dependent manner. These disturbances may not have obvious effects on the oestrus presence or duration but they could negatively affect the reproductive system of the goats. Further research regarding the direct or indirect effect of the aflatoxins on the reproductive system of the goats could interestingly be useful. Consequently, since aflatoxins are thought to be carcinogenic and teratogenic, the systematic control of the feedstuffs for the presence of AFB_1_ is strongly proposed.
